# Phytofabrication of Zinc Oxide Nanoparticles Using *Sida cordifolia* L. and Their Biomedical Applications

**DOI:** 10.1155/bca/6531769

**Published:** 2026-04-26

**Authors:** Mohammad Arham Siddiqui, Mohd Azam, Shabaaz Begum J. P., Sunil Kumar, Rajendra Prasad, Mohammad N. Alomary, Mohammad Azam Ansari, Hajed Obaid Abdullah Alharbi, Maryam Saleh Alhumaidi

**Affiliations:** ^1^ Department of Microbiology, Graphic Era (Deemed to be University), Dehradun, 248002, India, geu.ac.in; ^2^ Department of Medical Laboratories, College of Applied Medical Sciences, Qassim University, Buraydah, Qassim, 52571, Saudi Arabia, qu.edu.sa; ^3^ School of Agriculture, Uttaranchal University, Dehradun, Uttarakhand, 248007, India, uttaranchaluniversity.ac.in; ^4^ Advanced Diagnostic and Therapeutic Institute, King Abdulaziz City for Science and Technology, Riyadh, 11442, Saudi Arabia, kacst.edu.sa; ^5^ Department of Epidemic Diseases Research, Institute for Research and Medical Consultations (IRMC), Imam Abdulrahman Bin Faisal University, Dammam, 31441, Saudi Arabia, iau.edu.sa; ^6^ Department of Biology, College of Science, University of Hafr Al Batin, P.O. Box 1803 Hafr Al Batin, 31991, Kingdom of Saudi Arabia, uhb.edu.sa

**Keywords:** antibacterial activity, green chemistry, *Sida cordifolia*, sustainability, zinc oxide nanoparticles

## Abstract

Eco‐friendly approaches for nanoparticle synthesis have gained attention due to their sustainability and reduced environmental impact. Zinc oxide nanoparticles (ZnO NPs) exhibit versatile bioactivities, but conventional synthesis methods often involve toxic reagents. ZnO NPs were synthesized through a one‐step bioreduction process, utilizing the crude leaf extract of *Sida cordifolia* L. and employing the combustion method at 400°C. Characterization of the nanoparticles was analyzed through UV–Vis spectroscopy, FTIR, XRD, DLS, and SEM to assess structural and morphological properties. Antibacterial efficacy was tested through the resazurin plate assay, and antioxidant activity was evaluated through the hydrogen peroxide scavenging assay. UV–Vis shows a characteristic peak at ∼370 nm, which corresponds to the fundamental band gap transition of ZnO. FTIR and DLS verified organic moiety incorporation and nanoparticle size, respectively, while XRD confirmed the wurtzite structure. The average diameter of synthesized nanoparticles was between 88.5 and 90.2 nm. SEM provides detailed insights into their size, shape, surface morphology, and structural features. Functionalized nanoparticles demonstrated enhanced antibacterial efficacy against *E. coli*, *K. pneumoniae*, *S. aureus*, and *E. faecalis* with MICs of 12.5 μg/mL for Gram‐positive bacteria (*S. aureus* and *E. faecalis*), 100 μg/mL for *E. coli*, and 12.5 μg/mL for *K. pneumoniae*. Antioxidant activity shows concentration‐dependent radical scavenging. At a concentration of 3.12 μg/mL, ZnO NPs exhibited 28.7 ± 1.2% scavenging activity, which progressively increased to 64.3 ± 2.5% at 100 μg/mL. This one‐pot green synthesis provides a simple, scalable approach to biofunctionalized ZnO NPs with potent antibacterial and antioxidant properties, offering potential for biomedical applications.

## 1. Introduction

Nanotechnology, the manipulation of matter at the atomic and molecular scale (1–100 nm), has revolutionized fields such as medicine, energy, and environmental science due to the unique physicochemical properties of nanomaterials [1, 2]. Among the metal oxide nanoparticles, particularly zinc oxide nanoparticles (ZnO NPs), have derived significant attention for their optical, electrical, and antimicrobial properties [3, 4]. Traditional synthesis methods, however, often rely on toxic chemicals, high‐energy consumption, and generate hazardous byproducts, prompting a shift toward eco‐friendly alternatives [5, 6].

Green synthesis, which utilizes biological entities such as plant extracts and fungal or bacterial extracts, offers a sustainable, cost‐effective, and biocompatible route for nanoparticle fabrication [7, 8]. This approach aligns with the principles of green chemistry by minimizing waste and avoiding harmful reagents [9, 10].

*Sida cordifolia*, a medicinal plant rich in phytochemicals like flavonoids, alkaloids, and polyphenols [11], has emerged as a promising candidate for the green synthesis of ZnO NPs due to its bioactive compounds that act as reducing and stabilizing agents [12]. Zinc, a transition metal, is favored for nanoparticle synthesis owing to its abundance, low toxicity, and versatile applications in biomedicine (such as antimicrobial activities, drug delivery, antioxidant activity, and wound healing), catalysis, and environmental remediation [13, 14]. ZnO NPs exhibit a wide bandgap, strong UV absorption, and exceptional photocatalytic activity, making them ideal for antimicrobial coatings, solar cells, and pollution degradation [15, 16].

The green synthesis of ZnO NPs using *S. cordifolia* L. involves extracting plant metabolites to reduce zinc precursors, for example, zinc nitrate into nanoparticles, with parameters such as plant extract concentration, metal ion concentration, pH, temperature, and reaction time influencing size and morphology [12]. Characterization techniques are used to confirm the crystallinity, spherical morphology, and surface functionalization of phytofabricated ZnO NPs, which often outperform chemically synthesized counterparts in biocompatibility and catalytic efficiency [17, 18]. This method not only reduces environmental impact but also enhances the therapeutic potential of nanoparticles through synergistic effects with plant‐derived bioactive compounds [19, 20]. The integration of *S. cordifolia* L. in ZnO NP synthesis exemplifies the convergence of nanotechnology and sustainable practices, offering a scalable, eco‐friendly solution for advancing materials science and biomedical applications.

In the present work, the leaf extract of *S. cordifolia* L. was utilized for preparing ZnO NPs, and its antibacterial and antioxidant activities were evaluated. To our knowledge, this is the first study on ZnO NPs prepared from *S. cordifolia* L. leaf extract and its biomedical potential.

## 2. Materials and Methodology

### 2.1. Plant Material and Identification

The plant *S. cordifolia* L. was commonly available, and the leaves were collected from Kalagarh, District Pauri Garhwal, Uttarakhand, India (Lat 29.471226°, Long 78.788254°) as per guidelines issued by the Botanical Survey of India (BSI). The plant was identified by Dr. S. K. Singh, and the herbarium identification number is 2024‐2025/490, and the accession number is 1622.

### 2.2. Preparation of Plant Extract

The healthy leaves of *S. cordifolia* L. were collected from Kalagarh, Uttarakhand, India, and thoroughly washed with distilled water and shade dried under sterile conditions for 21 days. After that, the dried leaves were pulverized and sieved with a 16‐mm sieve and stored for the preparation of plant extract by using a Soxhlet apparatus. The dried leaf powder was taken in 1:1 with distilled water for the preparation, and the prepared extract was stored in a refrigerator at 4°C for further use.

### 2.3. Synthesis of Nanoparticles

Different volumes of *S. cordifolia* L. leaf extract have been taken for the green synthesis of ZnO NPs. 1 mM of zinc nitrate hexahydrate was taken in a porcelain crucible (30 mL), and the variable amounts of prepared leaf extract were also added. The solution was continuously agitated magnetically for 10–15 min at 70°C until the salt fully dissolved. After that, the crucible was transferred to the preheated muffle furnace for 10–20 min at 400°C for the process of combustion, in which the reaction mixture undergoes exothermic combustion accompanied by rapid gas evolution. Following this process, the resulting materials were subjected to calcination for 3 h at 400°C. Subsequently, the obtained nanoparticle powder was finely ground and employed in various biomedical applications [21].

### 2.4. Characterization of Phytosynthesized ZnO NPs

#### 2.4.1. Spectroscopic and Microscopic Analysis

Powdered X‐ray diffraction (PXRD) was utilized for the identification of phases using a Bruker AXS D8 Advance instrument with Cu Kal having a wavelength of 15.4 nm. UV–visible (UV–vis) absorption spectroscopy assessed their bandgap and adsorption properties, while Fourier transform infrared (FTIR) spectroscopy (PerkinElmer) was utilized to identify the vibrational modes of the nanoparticles’ chemical constituents. Dynamic light scattering (DLS) analysis was conducted using Malvern Zeta Version 7.11 to determine the hydrodynamic size distribution and zeta potential, confirming the nanoparticles’ stability in colloidal form. Scanning electron microscopy (SEM) was employed to examine surface morphology, size distribution, and aggregation state of the green‐synthesized ZnO NPs.

#### 2.4.2. Strains and Culturing of Microorganisms

The selected four microbial strains (*Klebsiella pneumoniae* ATCC13883, *Escherichia coli* MTCC68, *Enterococcus faecalis* ATCC29212, and *Staphylococcus aureus* MTCC737) were both Gram‐positive and Gram‐negative strains commonly implicated in human infections. Prior to the assay, each strain was streaked from frozen glycerol stocks onto appropriate solid media (e.g., Luria–Bertani agar for *E. coli* and *K. pneumoniae* and Mueller–Hinton agar for *S. aureus* and *E. faecalis*) and incubated at 35°C for 18 h to yield isolated colonies. A single colony was then inoculated into liquid broth and grown with shaking (120 rpm) at 37°C until the mid‐logarithmic phase (optical density at 600 nm ≈ 0.8). Cultures were standardized to ∼1 × 10^8^ CFU/mL (0.5 McFarland standard) and used immediately in antimicrobial susceptibility assays, ensuring uniform bacterial density and reproducible results across all four strains.

#### 2.4.3. Resazurin Microtiter Assay (REMA) Method

The REMA is a widely used, cost‐effective, and sensitive colorimetric method to assess the antibacterial activity of ZnO NPs. In this method, bacterial cells are cultured in microtiter plates and exposed to varying concentrations of ZnO NPs [21, 22]. Resazurin, a blue nonfluorescent dye, was then added to each well. Viable, metabolically active bacteria reduce resazurin into resorufin, which is pink and fluorescent. The intensity of the color change directly correlates with cell viability. If the ZnO NPs exert antibacterial effects, fewer bacteria remain metabolically active, leading to a lesser degree of color change or a retention of the blue color. This method not only provides a rapid screening tool but also helps in determining the minimum inhibitory concentration (MIC) and minimum bactericidal concentration (MBC) of the nanoparticles against different bacterial strains [23].

#### 2.4.4. Antioxidant Assay

In this method, the decay or loss of hydrogen peroxide is measured spectrophotometrically [24]. A solution of hydrogen peroxide (20 mM) was prepared in phosphate‐buffered saline (PBS, pH 7.4). Various concentrations of the sample or the standard in methanol were added to the hydrogen peroxide solution in PBS. After 10 min, the absorbance was measured at 230 nm.

## 3. Results

### 3.1. Characterization of ZnO NPs

#### 3.1.1. X‐Ray Diffraction (XRD)

**FIGURE 1:**
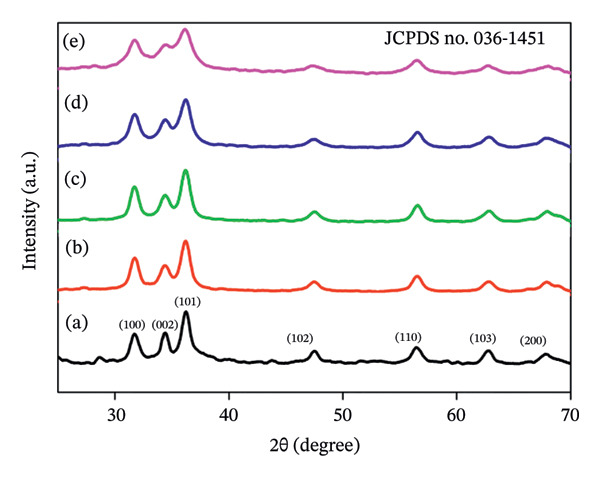
(a–e): Graph demonstrates the XRD pattern of ZnO NPs synthesized by utilizing 2, 4, 6, 8, and 10 mL of leaf extract, respectively.

Figures 1(a–e) illustrate the X‐ray diffraction profiles of ZnO NPs prepared with varying quantities of *S. cordifolia* L. leaf extract and confirms the formation of nanoparticles. The labeled diffraction peaks correspond closely to JCPDS card no. 036–1451. These Bragg diffraction peaks are consistent with the pure hexagonal wurtzite structure of ZnO (space group P63mc, no. 186) with lattice constants *a* = 3.24982 Å and *c* = 5.20661 Å. The average crystallite size found using the Debye–Scherrer equation was found to be ∼24 nm [21].

#### 3.1.2. Spectroscopic Analysis

**FIGURE 2:**
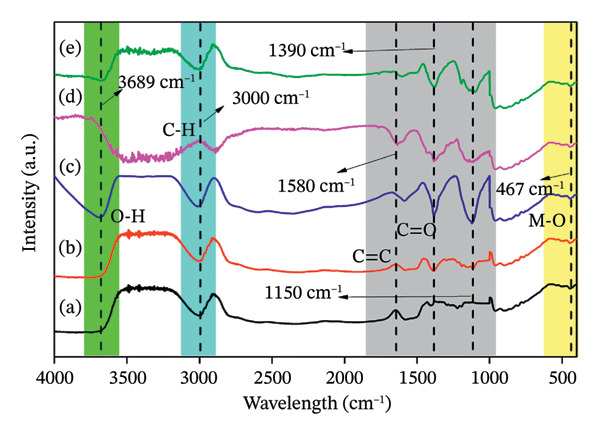
(a–e): FTIR spectra of ZnO nanoparticles prepared using 2, 4, 6, 8, and 10 mL of bioextract, respectively.

**FIGURE 3:**
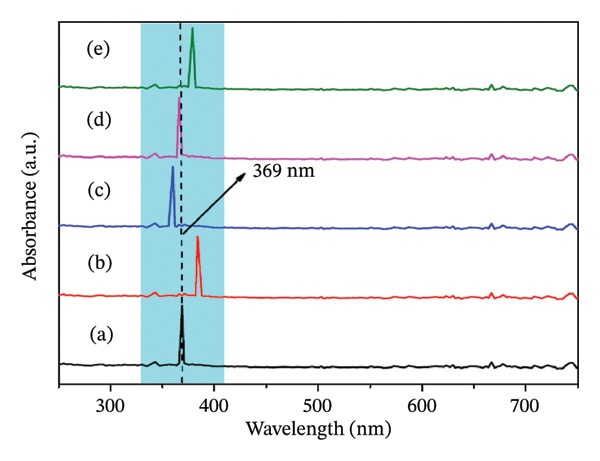
(a–e): UV–visible spectra ZnO nanoparticles prepared using 2, 4, 6, 8, and 10 mL of bioextract, respectively.

The characterization of ZnO NPs through UV–vis spectroscopy and FTIR spectroscopy FTIR provides valuable insights into their structural and optical properties. FTIR analysis is utilized to determine the active chemical moieties involved in the synthesis and stabilization of ZnO NPs. In the FTIR spectrum Figures 2(a–e), the characteristic Zn–O stretching vibrations typically appear between 400 and 600 cm^−1^, confirming the formation of ZnO NPs. Peaks around 3300–3500 cm^−1^ correspond to O–H stretching, indicating the presence of hydroxyl groups from the plant extract or water molecules on the nanoparticle surface. Additional peaks between 1500 and 1600 cm^−1^ suggest the presence of organic compounds such as flavonoids, phenols, or proteins, which act as capping and stabilizing agents during green synthesis. The peaks between 1000 and 1250 cm^−1^ show the presence of amide groups. UV–vis spectroscopy is widely used to analyze the optical properties and band gap of ZnO NPs. A strong absorption peak is typically observed between 350 and 380 nm, which corresponds to the intrinsic band gap transition of ZnO (Figures 3(a–e)) [21].

DLS analysis was employed to elucidate the hydrodynamic characteristics of green‐synthesized ZnO NPs derived from *S. cordifolia* L. leaf extract. Figures 4(a) and 4(b) depict the graphs of DLS showing the average diameter of phytofabricated ZnO NPs of concentration (a: 2 mL and b: 4 mL), respectively. Measurements were conducted at 25°C using a Malvern Zetasizer Nano ZS instrument, and the ZnO NPs of concentration 2 mL and 10 mL exhibited an average diameter of 88.5 and 90.2 nm, respectively, reflecting the combined contribution of the nanoparticle core and surface‐bound phytochemicals, while a PDI value of 0.2121 and 0.232, respectively, indicated a moderately narrow size distribution. These DLS outcomes substantiate the efficacy of the green synthesis protocol in yielding stable, monodisperse ZnO NPs suitable for biomedical applications.

FIGURE 4(a–b): Graphs of DLS depicting the average diameter of phytofabricated ZnO NPs of concentrations (a) 2 mL and (b) 10 mL, respectively.

**Figure:**
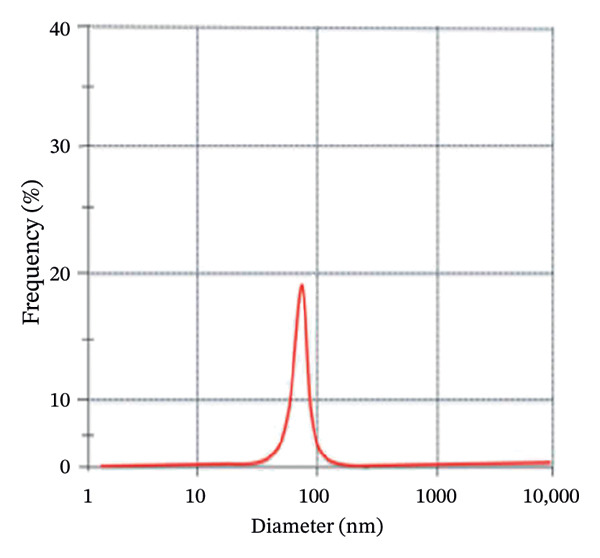
(a)

**Figure:**
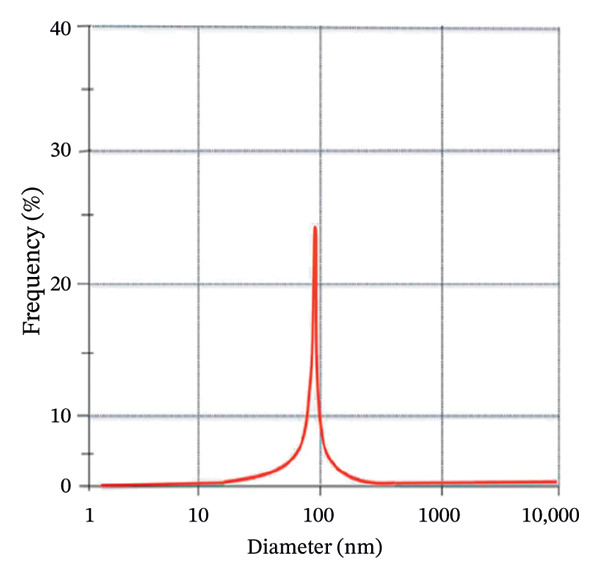
(b)

#### 3.1.3. Microscopic Analysis

**FIGURE 5:**
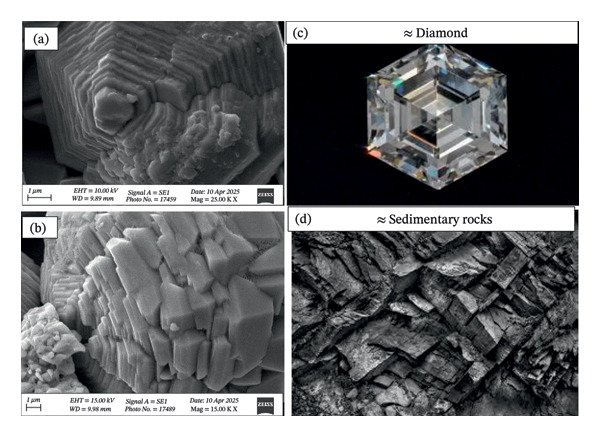
(a–d): SEM micrographs of ZnO nanostructures prepared using 2 mL (a–b) and 10 mL (c–d) of bioextract, respectively.

**FIGURE 7:**
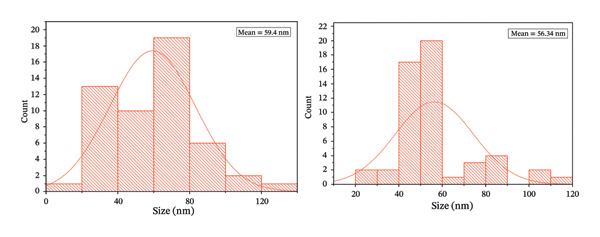
SEM‐based histogram with mean particle size and distribution of nanoparticles synthesized using 2 and 10 mL of leaf extract.

The characterization of ZnO NPs through microscopic analysis using SEM provides detailed insights into their size, shape, surface morphology, and structural features. SEM analysis is used to examine the surface morphology and overall particle size distribution of ZnO NPs. It provides high‐resolution images that reveal the texture, agglomeration state, and uniformity of the nanoparticles. Figures 5(a), 5(b), 5(c), and 5(d) show the SEM micrographs of ZnO nanostructures prepared using 2 mL (a–b) and 10 mL (c–d) of bioextract, respectively. ZnO NPs typically exhibit hexagonal wurtzite structures. The SEM images also confirm the degree of agglomeration and surface roughness, which influence the functional properties of ZnO NPs, and because of the confirmed size, further assays such as antibacterial and antioxidant assays were conducted using ZnO NPs synthesized using 2 mL and 10 mL plant extract concentrations [21]. The EDS spectrum, Figures 6(a) and 6(b) exhibited prominent peaks corresponding to Zn (Lα at ∼1.0 keV) and O (Kα at ∼0.52 keV), confirming the formation of ZnO NPs. A minor carbon peak was also observed, which may be attributed to phytochemical residues from the plant extract used during green synthesis. The absence of additional impurity peaks indicates the high purity of the synthesized material. The particle size distribution histogram, Figure 7, indicates the mean size of 59.40 and 56.34 nm, respectively, for 2 and 10 mL volume of leaf extract utilized in the synthesis of nanoparticles.

FIGURE 6(a–b): EDS elemental analysis graphs depict the purity of the phytosynthesized ZnO NPs.

**Figure:**
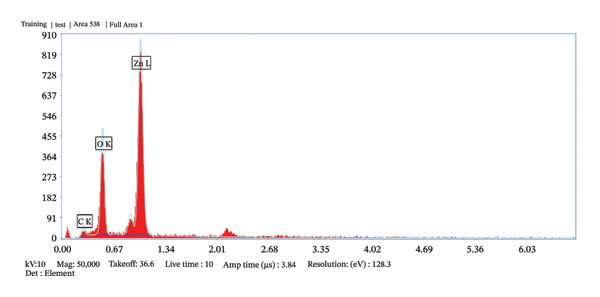
(a)

**Figure:**
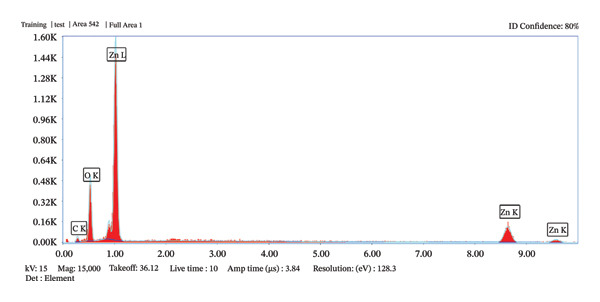
(b)

### 3.2. Antibacterial Activity

**FIGURE 8:**
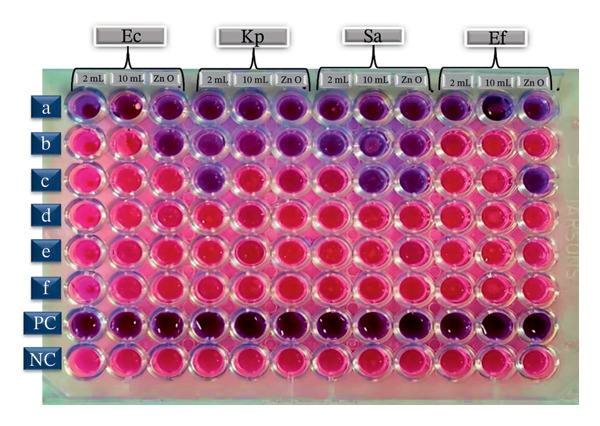
Antibacterial activity of ZnO NPs synthesized using different volumes of leaf extract (2 mL and 10 mL) and commercially available ZnO NPs. Concentrations (a–f: 100, 50, 25, 12.5, 6.25, and 3.12 μg/mL) show the twofold dilution of ZnO NPs. Microtiter plates depicting MIC and MBC against Ec: *Escherichia coli*, Kp: *Klebsiella pneumoniae*, Sa: *Staphylococcus aureus*, and Ef: *Enterococcus faecalis* pathogenic bacterial strains.

This study was conducted to assess the antibacterial efficacy of eco‐friendly fabricated ZnO NPs against a range of disease‐causing bacterial species in humans. The MIC denotes the smallest amount of a bioactive compound needed to inhibit observable microbial proliferation, whereas the MBC indicates the minimal quantity essential to eliminate the microorganisms, evaluated through the microdilution technique in broth medium, as shown in Figure 8. Also, Figures 9(a) and 9(b) demonstrate the graphical comparison between MIC and MBC of green‐synthesized ZnO NPs of different volumes (a: 2 mL and b: 10 mL) and commercially available ZnO NPs.

FIGURE 9(a–b): Graph demonstrates the comparison between MIC and MBC of green‐synthesized ZnO NPs of different concentrations ((a) 2 mL and (b) 10 mL) and commercially available ZnO NPs.

**Figure:**
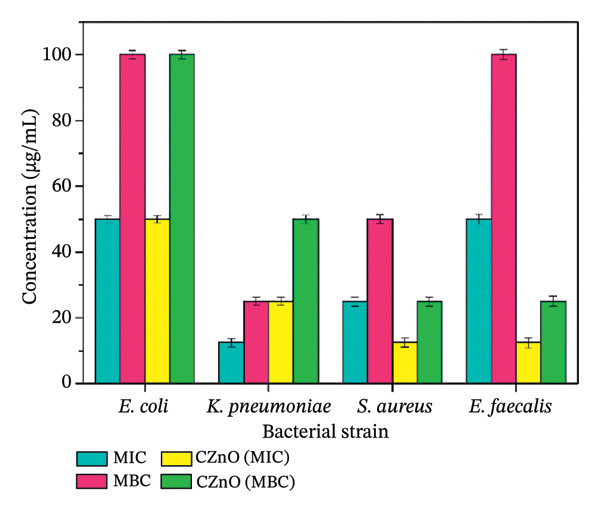
(a)

**Figure:**
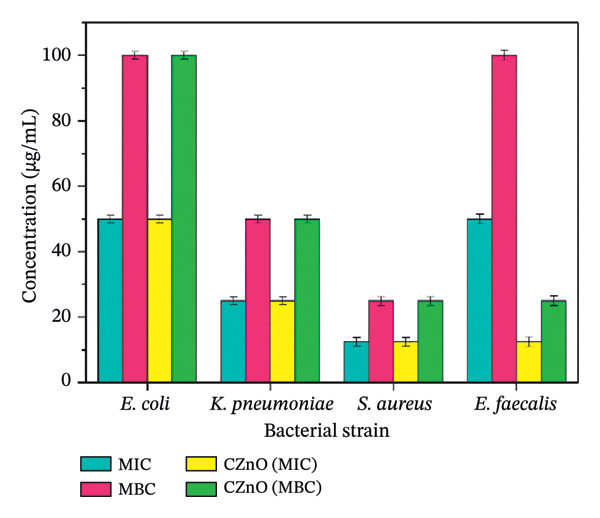
(b)

MIC and MBC readings of plant‐mediated ZnO NPs targeting the tested pathogens are presented in Table 1. The phytofabricated ZnO NPs demonstrated significant antibacterial effects against *K. pneumoniae, E. coli, E. faecalis*, and *S. aureus*, with varying degrees of sensitivity observed. The MIC of ZnO NPs determined for Gram‐positive strains (*S. aureus* and *E. faecalis*) was 12.5 μg/mL. For Gram‐negative strains (*E. coli* and *K. pneumoniae*), the MIC values were 100 and 12.5 μg/mL, respectively. These results demonstrate that both Gram‐positive and Gram‐negative bacterial strains exhibit sensitivity to ZnO NPs synthesized through green methods. The study conducted by Wahab et al. [25] also shows the same pattern, as the investigation reveals that the lowest concentration of ZnO NPs solution inhibiting the growth of microbial strains is found to be 5 μg/mL for *K. pneumoniae*, whereas for *E. coli*, *S. aureus*, and *S. typhimurium*, it was calculated to be 15 μg/mL. The variation in antibacterial activity between Gram‐positive and Gram‐negative bacteria is mainly due to differences in their cell wall structures. Although Gram‐negative bacteria have an additional outer membrane that limits permeability, ZnO NPs exert their effect largely through ROS generation and membrane damage, which can bypass these structural barriers. Thus, nanoparticle properties and strain‐specific oxidative stress resistance may influence susceptibility more strongly than Gram classification alone [26].

**TABLE 1:** Antibacterial effect of phytofabricated ZnO NPs from different concentrations of botanical extract against selected pathogenic bacterial strains (concentration expressed in μg/mL).

**Nanoparticles**	**Strains**				
**ZnO NPs**		***E. coli* (μg/mL)**	***K. pneumoniae* (μg/mL)**	***S. aureus* (μg/mL)**	***E. faecalis* (μg/mL)**

2 mL	MIC	50 ± 1.62	12.5 ± 1.68	25 ± 1.65	50 ± 1.62
	MBC	100 ± 1.68	25 ± 1.62	50 ± 1.68	100 ± 1.65

10 mL	MIC	50 ± 1.65	25 ± 1.62	12.5 ± 1.64	50 ± 1.68
	MBC	100 ± 1.62	50 ± 1.65	25 ± 1.68	100 ± 1.64

CZnO	MIC	50 ± 1.68	25 ± 1.62	12.5 ± 1.62	12.5 ± 1.65
	MBC	100 ± 1.62	50 ± 1.69	25 ± 1.65	25 ± 1.68

### 3.3. Antioxidant Assay

The free radical scavenging ability of plant‐mediated ZnO NPs derived from *S. cordifolia* leaf extract was evaluated using the hydrogen peroxide (H_2_O_2_) scavenging assay. The results demonstrated a concentration‐dependent increase in the scavenging activity of ZnO NPs. Figures 10(a), 10(b), and 10(c) show the antioxidant activity of biosynthesized ZnO NPS with different concentrations (a: 2 mL, b: 10 mL, and c: commercially available ZnO NPs).

FIGURE 10(a‐c): Antioxidant activity of biosynthesized ZnO nanoparticles with different concentrations: (a) 2 mL, (b) 10 mL, and (c) commercially available ZnO NPs.

**Figure:**
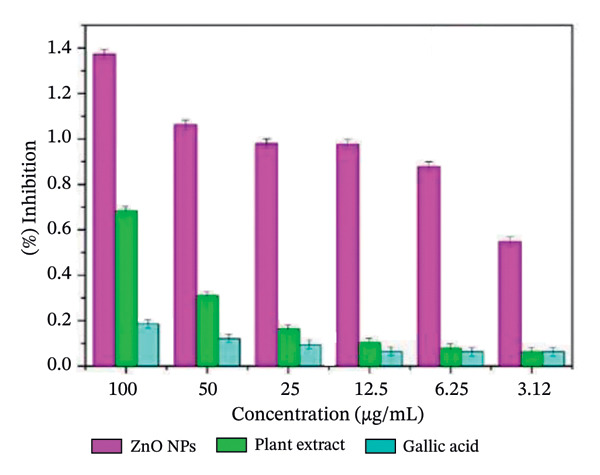
(a)

**Figure:**
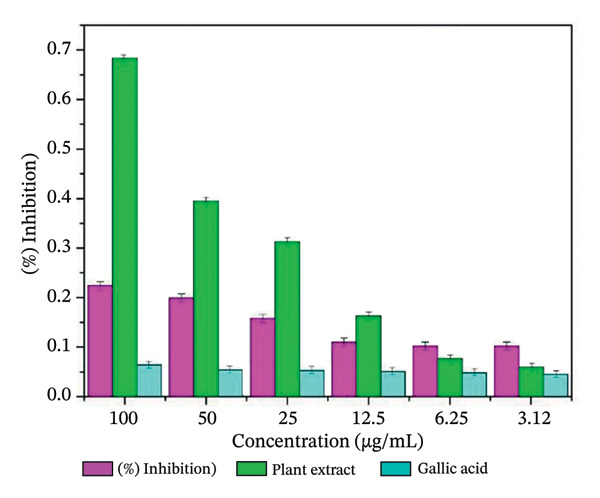
(b)

**Figure:**
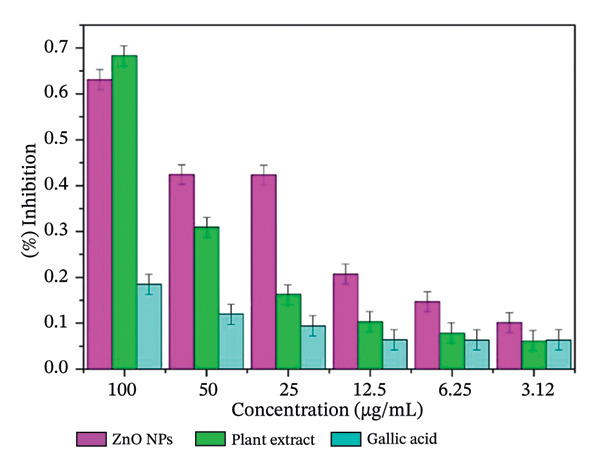
(c)

At a concentration of 3.12 μg/mL, the ZnO NPs exhibited 28.7 ± 1.2% scavenging activity, which progressively increased to 64.3 ± 2.5% at 100 μg/mL. In comparison, the standard gallic acid showed 76.8 ± 1.6% scavenging activity at the same concentration. The IC_50_ value of ZnO NPs was found to be 57.4 μg/mL, whereas that of gallic acid was 36.9 μg/mL, indicating a moderate antioxidant potential of the synthesized nanoparticles.

The observed activity may be attributed to the presence of phytochemicals from *S. cordifolia* leaf extract capping the surface of ZnO NPs, which possibly enhance free radical neutralization. These results suggest that ZnO NPs synthesized via *S. cordifolia* leaf extract exhibit promising antioxidant properties, which could be beneficial in biomedical and therapeutic applications.

## 4. Mechanism of Action of ZnO NPs

ZnO NPs have attracted significant attention due to their broad‐spectrum applications in biomedicine, environmental remediation, and industry [27–29]. Their mechanism of action is complex and multifactorial, involving a combination of physical interactions, chemical reactivity, and biological responses [13].

**FIGURE 11:**
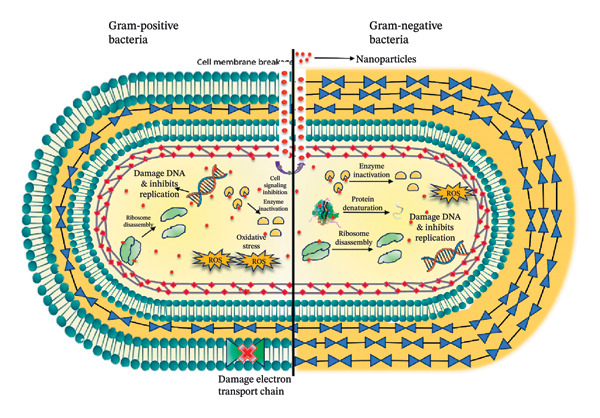
Proposed mechanism of action of phytosynthesized zinc oxide nanoparticles.

One of the primary mechanisms of action of ZnO NPs is the generation of reactive oxygen species (ROS) [30–32]. When ZnO NPs are exposed to light or even under dark conditions, they can catalyze the formation of ROS such as hydroxyl radicals (•OH), superoxide anions (O_2_•^−^), and hydrogen peroxide (H_2_O_2_) [33]. The production of these ROS is largely attributed to the semiconductor nature of ZnO. Under UV light, electrons in the valence band of ZnO get excited and are transferred to the conduction band. These charge carriers then interact with oxygen and water molecules in the environment, leading to the formation of various ROS. The generated ROS are highly reactive and can damage key cellular components such as lipids, proteins, and nucleic acids [34, 35]. This oxidative stress disrupts normal cellular functions, leading to cell membrane damage, protein denaturation, and DNA fragmentation, which eventually causes cell death [36, 37]. In microbial cells, such oxidative stress can result in the disruption of vital metabolic pathways and the inactivation of enzymes critical for survival. The proposed mechanism of action of phytosynthesized ZnO NPs is shown in Figure 11.

Another important mechanism is the release of zinc ions (Zn^2+^) from the ZnO NPs. The dissolution of ZnO in biological fluids or acidic microenvironments (such as those found in infection sites or within cancer cells) results in the release of Zn^2+^. These ions can interfere with the cellular machinery in multiple ways. Zinc ions are known to bind to proteins and enzymes, altering their structure and function [38, 39]. This ion‐mediated interaction can disrupt key metabolic processes and lead to enzyme inhibition, further contributing to the cytotoxic effects on microbial or cancer cells. Moreover, the accumulation of zinc ions can disturb the ionic balance within the cell, potentially triggering stress responses and apoptosis [40]. Due to their nanoscale dimensions and high surface‐to‐volume ratio, ZnO NPs can adhere to and even penetrate microbial cell membranes [41]. The nanoparticle surfaces can interact with membrane phospholipids, leading to structural changes or even perforations in the cell membrane [42]. The combined effects of membrane disruption, oxidative stress, and zinc ion release create a hostile environment that significantly impairs cellular function.

## 5. Conclusion

This study represents the first account of using varying volumes of *S. cordifolia* leaf extract as a reducing agent in the phytosynthesis of ZnO NPs. Characterization confirmed the formation of polydisperse ZnO NPs. These particles demonstrated strong antimicrobial activity against selected human pathogens, showing greater efficacy toward both Gram‐positive and Gram‐negative bacteria. These nanoparticles also show potent antioxidant activity and may prove advantageous for medical and therapeutic uses. Their pronounced abrasive nature likely promoted the disruption of microbial cell membranes, thereby enhancing both their bactericidal and cytotoxic effects.

## Funding

The researchers would like to thank the Deanship of Graduate Studies and Scientific Research at Qassim University for financial support (QU‐APC‐2026).

## Disclosure

All authors read and approved the manuscript for publication.

## Conflicts of Interest

The authors declare no conflicts of interest.

## Data Availability

The data that support the findings of this study are available upon request from the corresponding author. The data are not publicly available due to privacy or ethical restrictions.
